# Ligand Migration from Cluster to Support: A Crucial Factor for Catalysis by Thiolate‐protected Gold Clusters

**DOI:** 10.1002/cctc.201801474

**Published:** 2018-11-26

**Authors:** Bei Zhang, Annelies Sels, Giovanni Salassa, Stephan Pollitt, Vera Truttmann, Christoph Rameshan, Jordi Llorca, Wojciech Olszewski, Günther Rupprechter, Thomas Bürgi, Noelia Barrabés

**Affiliations:** ^1^ Department of Physical Chemistry University of Geneva Quai Ernest-Ansermet 30 CH-1211 Geneva Switzerland; ^2^ Institute of Materials Chemistry Technische Universität Wien Getreidemarkt 9/BC/01 1060 Vienna Austria; ^3^ Institute of Energy Technologies, Dep. of Chemical Engineering and Barcelona Research Center in Multiscale Science and Engineering Universitat Politècnica de Catalunya EEBE Eduard Maristany 16 08019 Barcelona Spain; ^4^ ALBA Synchrotron Light Facility Carrer de la Llum 2–26 08290 Cerdanyola del Vallès Barcelona Spain; ^5^ Faculty of Physics University of Bialystok 1 L K. Ciolkowskiego Str. 15-245 Bialystok Poland

**Keywords:** ligand effect, nanocluster, gold, heterogeneous catalysis, X-ray Absorption

## Abstract

Thiolate protected metal clusters are valuable precursors for the design of tailored nanosized catalysts. Their performance can be tuned precisely at atomic level, e. g. by the configuration/type of ligands or by partial/complete removal of the ligand shell through controlled pre‐treatment steps. However, the interaction between the ligand shell and the oxide support, as well as ligand removal by oxidative pre‐treatment, are still poorly understood. Typically, it was assumed that the thiolate ligands are simply converted into SO_2_, CO_2_ and H_2_O. Herein, we report the first detailed observation of sulfur ligand migration from Au to the oxide support upon deposition and oxidative pre‐treatment, employing mainly S K‐edge XANES. Consequently, thiolate ligand migration not only produces clean Au cluster surfaces but also the surrounding oxide support is modified by sulfur‐containing species, with pronounced effects on catalytic properties.

Small metal nanoparticles and clusters are well known for their high catalytic activity.^1^ Nanoscale particles can be stabilized by protecting ligands and support materials, both having a strong influence on their catalytic properties.^2^

Typical synthetic procedures involve the use of ligands for obtaining a defined particle size, shape and structure. To improve their stability during catalytic applications, the ligand‐protected clusters are supported on different oxide materials. However, strongly coordinating ligands may block the active Au sites and/or alter their electronic properties.2c Therefore, different treatments^3^ are typically applied to remove the protecting ligands, assuming complete removal of the ligands via gas phase SO_2_, CO_2_ and H_2_O. Previous thermal activation studies of ligand‐protected metal clusters have focused on alterations of size and structure upon different treatments.^4^ The exact fate of the thiolate ligands was not considered so far.

Thiolate protected Au nanoclusters in the size range from sub‐nanometer to 2 nm have shown extraordinary catalytic selectivity and activity, which depends on their size^5^ and structure.^6^ Au nanoclusters are composed of a dense gold core and protecting S‐(Au−S)_n_ (n=1, 2) units (staples).^7^ Supported Au clusters were shown to be active for several reactions, such as CO oxidation,^8^ cyclohexane5b,^9^ aerobic alcohol^10^ and styrene oxidation.^11^ It has been observed that the thiolate ligands play an essential role2c in the catalytic activity of Au nanoclusters in gas8b and liquid phase^10^ reactions. The ligand coverage around the Au cluster core affects the activity and selectivity, which has been ascribed to electronic and steric effects, but this has been controversially discussed.

Supported ligand protected clusters (Au_x_(SR)_y_/CeO_2_, x=25, 38 and 144), were found to be active in reactions such as CO oxidation, even without removing any ligands.8a,^12^ For Au_25_(SR)_18_/CeO_2_, it was reported that the thiol ligands acted as a double‐edged sword, stabilizing on the one hand the Au cluster structure but on the other hand blocking CO adsorption on Au sites.8b Therefore, a partial removal of thiolate ligands was typically required for CO oxidation.8b In the case of liquid phase aerobic oxidation of benzyl alcohol, Au_25_(SC_12_H_25_)_18_ supported on porous carbon nanosheets showed no activity with the complete ligand shell, but only when the ligands were partly removed. The ligand coverage also affects selectivity, since thiolates reduce the oxidation ability of Au by withdrawing electrons, but also by inducing site isolation.^10^

Despite the well‐documented importance of ligand removal for catalyst activation, the exact reaction pathways have not yet been considered in detail. As mentioned, it was assumed that the ligands are removed to the gas phase and their fate has been completely neglected up to now.^13^

S K‐edge XAFS was used in this work to directly follow the evolution of the thiolate ligands upon Au_38_(SR)_24_ cluster deposition on CeO_2_. Clear evidence of ligand migration from the gold cluster to the support was obtained, manifested by formation of unexpected oxidized sulfur species on the support. The detected SO_x_ compounds increased and evolved upon thermal treatments. The redistribution and oxidation of the ligands modified the surface, a factor that can alter the catalyst properties. It may explain the different catalytic performances depending on the degree of ligand removal in different reactions.8a,8b,^9^,^10^,^12^ In our previous work on supported Au_38_(SR)_24_ clusters, unexpected cyclohexanethiol was obtained among the products of cyclohexane oxidation.^9^ The only possible source of sulfur in the reaction media were the cluster ligands, thus clearly evidencing the active role of the thiolate ligands in the reaction.^9^ Figure 1 shows S K‐edge (2472 eV) XAFS spectra of Au_38_(SR)_24_ clusters supported on CeO_2_. Identification of the S species in the samples was carried out by comparing the XANES spectra with S reference compounds (oxidation states: −1, 0, +3, +4 and +6, Figure 1b). Unsupported Au_38_(SR)_24_ clusters were also measured as reference, showing a pre‐edge feature at 2471 eV related to S−Au bonds and a S−C peak at 2473 eV, in agreement with previously reported measurements.^14^

**Figure 1:**
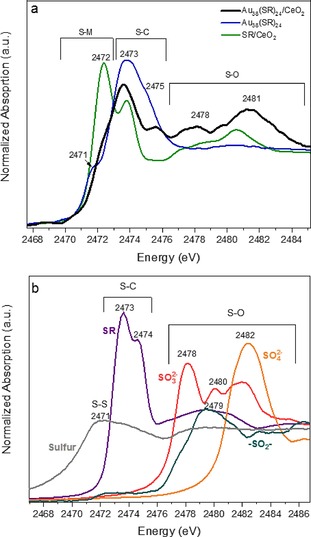
a: XANES spectra at S K‐edge of Au_38_/CeO_2_ catalysts, the unsupported cluster Au_38_(SC_2_H_4_Ph)_18_ and the thiol ligand supported on CeO_2_ are also included as references; b: XANES spectra at S K‐edge of different reference materials for comparison.

Once the clusters are supported on CeO_2_, the S−Au to S−C peak intensity decreases, indicating partial detachment of thiolate ligands from the Au nanoclusters. In addition, unexpected peaks between 2478 and 2486 eV appeared, related to S species in high oxidation states (disulfide, SO_3_^2−^ and SO_4_^2−^). The only possible source of S are the thiolate ligands, denoting ligand migration to the oxide material upon supporting. Both atmospheric and support lattice oxygen may contribute to the formation of these high oxidation state S species. The peak close to 2476 eV could be attributed to adsorbed disulfides on Ce atoms, based on previous reports,^15^ whereas the S−O peaks at 2478 and 2481 eV are due to SO_3_^2−^ and SO_4_^2−^.15a,^16^ Different interactions between the ligands and support sites lead to these distinct oxidized S species. The SR/CeO_2_ reference spectra in Figure 1a (2472 eV due to S−Ce bond) confirmed this picture. In this case, the ligand phenylethanethiol (SHC_2_H_4_Ph) was deposited on CeO_2_ as blank sample. Comparing the spectra of the supported and unsupported ligands (Figs. 1a,b), new bands in the region around 2478 and 2482 eV, characteristic of S−O bonds, emerged upon supporting. Furthermore, different double peaks appeared at the white‐line, characteristic of thiosulfates, organic disulfides, etc., related to 1 s transitions in S−C or S−H bonds. A clear white‐line shift to lower energy also occurred upon supporting the ligand, which may be attributed to S−Ce interaction. Thus, this represents the first direct evidence of the redistribution of S species between gold and metal oxide upon supporting the clusters, an effect that has completely been neglected up to now.

Deposition on Al_2_O_3_, another frequently used support material, was also studied, leading to different SO_x_ species. For Au_38_(SR)_24_/Al_2_O_3_, S−O peaks at 2482 and 2498 eV were found, denoting the presence of SO_3_^2−^ and SO_4_^2−^ (Figure S3). The interactions of sulfur compounds with oxides such as Al_2_O_3_ and CeO_2_ are relevant for catalyst poisoning or desulfurization processes.^17^

The reactivity of S‐containing molecules on oxides was found to be linked to properties such as acidity‐basicity, pore structure, band gaps, and oxygen vacancies.17c The efficiency to oxidize various aromatic and heterocyclic thiols to disulfides increases upon addition of Au nanoparticles.17d This may be related to the strong interaction of the thiol ligands with the oxide support, as observed by the S K‐edge measurements.

The evolution of the S species upon thermal treatment was followed by S K‐edge XANES (Figure 2). Increasing the temperature, thiolate ligands detached from the Au clusters and the ligand carbon backbone broke, resulting in the decrease of the peaks between 2471 and 2475 eV (S−C). This is in agreement with our previous Au L_3_‐edge EXAFS experiments, in which Au−S bonds clearly decreased during thermal treatments (whereas the Au core structure was maintained (Figure S4)).^9^ The sulfur oxide species increased (2478–2482 eV) and further oxidation to SO_4_^2−^ took place, as evidenced by the increase and shift to higher energies of the peak around 2480 eV. An attempt to estimate the amount of species at every step was performed using reference compounds. Qualitative linear combination was done. The analysis is complicated by the double peaks and shoulders at the white‐line, which may induce considerable errors in quantification. The general tendency is displayed in Figure S5, with the main component SO_3_^2−^ decreasing and turning into SO_4_^2−^. The contribution of Au_38_(SR)_24_ has a major drop from 150 °C to 250 °C, associated with the breaking S−Au bonds. At the same time, the SR/CeO_2_ portion just shows a slight decrease.

**Figure 2:**
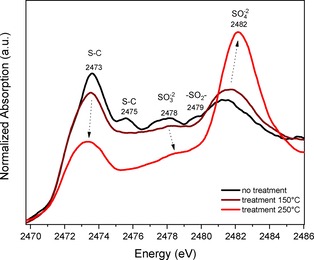
XANES spectra at S K‐edge of Au_38_/CeO_2_ catalysts, fresh and treated at 150 °C and 250 °C under oxygen atmosphere. Reference compound spectra are also included in Figure 1b.

STEM‐HAADF with EDX analysis of Au_38_(SR)_24_/CeO_2_ pretreated in O_2_ at 150 °C was performed to determine microstructure and surface composition (Figure 3). The ceria support crystallites exhibited a fairly wide distribution of sizes, ranging approximately from 10 to 50 nm. Given the high atomic number of Ce and the small size of the Au nanoclusters, clear discrimination was not obtained. In addition, due to the small dimensions of the Au nanoclusters (around 1 nm) and their high dispersion over the ceria support, EDX spectra from a single Au cluster could not be obtained. The EDX spectrum recorded over the ceria support areas (Figure 3a) did not show S. In contrast, the EDX spectrum of areas containing Au clusters (Figure 3b) revealed the presence of S, confirming that S is located near the Au clusters.

**Figure 3:**
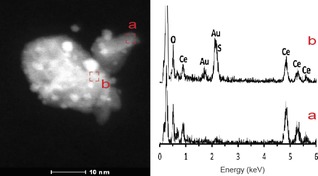
HAADF‐STEM images of Au_38_/CeO_2_ catalysts treated in O_2_ at 150 °C and EDX analysis of the area marked in the inset.

Figure 4 shows the XPS spectra of Au 4 f/2 and S 2p regions (including peak fitting) of Au_38_(SR)_24_/CeO_2_ after 150 °C pretreatment. The Au 4 f 7/2 signal at 84.6 eV can be attributed to metallic gold, with its binding energy being around ∼0.6 eV higher than that of bulk metallic Au.^18^ Huang et al. reported similar binding energies for Au nanoclusters supported on ceria.^19^ Also, Zhang et al. showed that for Au_n_(SR)_m_ nanoparticles of decreasing size, the Au 4 f peaks shifted to higher binding energy,^20^ resulting from a nanosize effect and from surface metal−ligand interactions.^20^ In the S 2p spectra (Figure 4), two components at 161.3 and 167.4 eV were present. The peak at 161.3 eV can be attributed to the S−Au bond, in agreement with values for metal sulfides^21^ or with the S−Au bond in self‐assembled monolayers.^22^ The peak at 167.4 eV is assigned to fully oxidized sulfur, consistent with the observation of SO_3_^2−^ and SO_4_^2−^ by XANES. This also agrees with XPS results of Devillers et al., who reported oxidation of sulfur of self‐assembled monolayers in an oxygen environment.^23^ Pookpanratana et al. also reported fully oxidized sulfur at rather high binding energies of ∼168.6 eV.^24^

**Figure 4:**
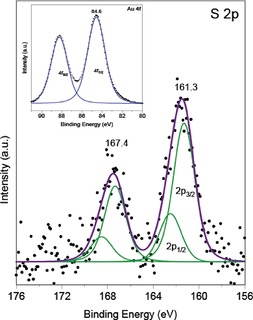
XPS spectra of the S 2p3 and Au 4 f (inset) core levels of the Au_38_(SR)_24_/CeO_2_ treated at 150 °C.

The migration and redistribution of S species, as reported here, strongly depends on the pretreatment conditions (and type of oxide support) and play an important role in catalysis. Thus, we anticipate a complex behavior of selectivity resulting from the evolution of S species as a function of pretreatment and during catalysis. This was already observed in our previous studies on cyclohexane oxidation,^9^ which indicated a strong dependence of selectivity on the pretreatment conditions. The redistribution of S species may affect the oxidation reaction possibly in two ways: 1) direct participation of the S species adsorbed on the support in the reaction, 2) poisoning of catalytically active sites on the support surface and affecting the charge transfer between support and nanocluster.

The adsorption of sulfur species (either on metal sites or oxygen vacancies) changes the charge distribution on the surface, which is an important factor for the activation of O_2_. Jin and coworkers, for example, reported an enhanced catalytic activity of Au_38_(SR)_24_/CeO_2_ catalyst in CO oxidation by increasing the Ce^3+^/Ce^4+^ ratio of the support.8b,^12^,^25^ Recently, when comparing different bulky thiol ligands, differences in catalytic activity were ascribed just to interface effects, but without giving further details.^26^ This clearly shows the importance of the support electron density on catalysis and underlines the importance of our finding. The thiol migration and formation of oxidized sulfur species certainly changes the electron density on the support. Indeed, perspective review publications have highlighted the need to better understand the important ligand effects on the structure and catalytic properties of supported metal clusters.1b,^27^

In summary, we have shown that upon supporting thiolate protected gold clusters, the thiolates are redistributed between cluster and support, leading to oxidized sulfur species that alter the electronic and adsorption properties of the support (Scheme 1). This effect has completely been neglected up to now but we believe that it must be taken into account to fully understand the complex selectivity patterns of supported thiolate‐protected clusters in catalysis.

**Scheme 1:**
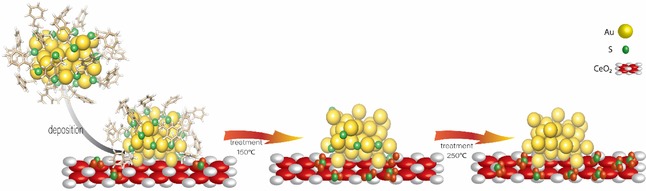
Thiolate ligands evolution upon cluster deposition on CeO_2_ and oxidative treatments.

## **Experimental Section**

**Synthesis of Au_38_/M_x_O_y_ catalysts**. Thermally treated Au_38_/M_x_O_y_ were prepared as described in the previous report.^9^ Au_38_(SC_2_H_4_Ph)_24_ is denoted as Au_38_(SR)_24_. Detailed description of the cluster synthesis, catalysts preparation and characterization can be found in the Supporting Information

**XAFS studies**. X‐ray absorption fine structure (XAFS) measurements at the S K‐edge (2.4720 keV) and Au L_3_‐edge (11.9187 keV) were carried out at the BL22‐CLAESS beamline at the ALBA synchrotron (Barcelona, Spain) for fresh, thermally pretreated and used catalysts. The samples were prepared as 5 mm pellets and mounted on the beamline sample holder. Measurements at both S K‐edge and Au L_3_‐edge were performed in fluorescence mode under vacuum and low temperature conditions (liquid nitrogen T≈80 K). Sulfur reference compounds (sulfur, sulfone, Na_2_SO_3_, Na_2_SO_4_, corresponding oxidation states: 0, +3, +4, and +6; and Al_2_O_3_ and CeO_2_ supported phenylethanethiol) were also measured. Additional XAFS measurements at Au L_3_‐edge for selected CeO_2_ supported catalysts were carried out at the SuperXAS beamline at the Swiss Light Source (Villigen, Switzerland). Fluorescence signal was detected with a five‐element SDD detector (SGX). The powder samples were placed in a quartz capillary and cooled down with a cryo‐gun to liquid nitrogen temperature. The data analysis was performed according to standard procedures using Ifeffit software.^28^

## Conflict of interest

The authors declare no conflict of interest.

## Supporting information

As a service to our authors and readers, this journal provides supporting information supplied by the authors. Such materials are peer reviewed and may be re‐organized for online delivery, but are not copy‐edited or typeset. Technical support issues arising from supporting information (other than missing files) should be addressed to the authors.

Supplementary
