# Supplementary material for: Ligand Migration from Cluster to Support: A Crucial Factor for Catalysis by Thiolate‐protected Gold Clusters
Source: ChemCatChem. 2018 Nov 26;10(23):5372–6. doi: 10.1002/cctc.201801474 (PMC6348379; doi:10.1002/cctc.201801474)
Supplement: Supplementary file 1 — Supplementary [file CCTC-10-5372-s001.pdf]

Supporting Information

© Copyright Wiley-VCH Verlag GmbH & Co. KGaA, 69451 Weinheim, 2018

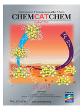

# Ligand Migration from Cluster to Support: A Crucial Factor for Catalysis by Thiolate-protected Gold Clusters

Bei Zhang, Annelies Sels, Giovanni Salassa, Stephan Pollitt, Vera Truttmann, Christoph Rameshan, Jordi Llorca, Wojciech Olszewski, Günther Rupprechter, Thomas Bürgi,* and Noelia Barrabés*© 2018 The Authors. Published by Wiley-VCH Verlag GmbH & Co. KGaA. This is an open access article under the terms of the Creative Commons Attribution License, which permits use, distribution and reproduction in any medium, provided the original work is properly cited.

Supporting Information
©Wiley-VCH 2016
69451 Weinheim, Germany

# Ligand migration from cluster to support: A crucial factor for catalysis by thiolate-protected gold clusters

Bei Zhang[a], Annelies Sels[a], Giovanni Salassa[a], Stephan Pollitt[b],Vera Truttmann[b], Christoph Rameshan[b], Jordi Llorca[c], Wojciech Olszewski[d,e], Günther Rupprechter[b],Thomas Bürgi [a]* and Noelia Barrabés [b]*

[a] Dr. Bei Zhang, Annelies Sels, Dr. Giovanni Salassa, Prof.Thomas Bürgi
Department of Physical Chemistry
University of Geneva
Quai Ernest-Ansermet 30, CH-1211 Geneva, Switzerland
E-mail: Thomas.buergi@unige.ch

[b] Stephan Pollitt, Vera Truttmann, Dr. Christoph Rameshan, Prof. Günther Rupprechter, Dr. Noelia Barrabés
Institute of Materials Chemistry
Technical University of Vienna
Getreidemarkt 9/BC/01, 1060 Vienna, Austria
E-mail: Noelia.rabanal@tuwien.ac.at

[c] Prof. Jordi Llorca
Institute of Energy Technologies, Dep. of Chemical Engineering and Barcelona Research Center in Multiscale Science and Engineering
Universitat Politècnica de Catalunya
EEBE, Eduard Maristany 16, 08019 Barcelona, Spain

[d,e] Dr. Wojciech Olszewski
ALBA Synchrotron Light Facility
Carrer de la Llum 2-26, 08290 Cerdanyola del Vallès, Barcelona, Spain
Faculty of Physics
University of Bialystok
1L K. Ciolkowskiego Str., 15-245 Bialystok, Poland

Supporting information for this article is given via a link at the end of

## Experimental Details

**Chemicals :** Tetrachloroauric acid trihydrate (Aldrich, 99.9+ %), L-glutathione (reduced, Sigma-Aldrich, > 99 %), sodium borohydride (Fluka, > 96 %), 2-phenylethanethiol (Aldrich, 98 %), methanol (VWR, > 99.8 %), acetone (Fluka, > 99.5 %), methylene chloride (Sigma-Aldrich, > 99.9 %), toluene (Sigma-Aldrich, 99.9 %), PTFE syringe filters (0.2 µm, Carl Roth, Karlsruhe/Germany), BioBeads S-X1 (BioRad) for SEC separation. Nanopure water (miliQ) (> 18 MΩ) was used.

**Synthesis $Au_{38}(SC_2H_4Ph)_{24}$ Nanoclusters.:** The synthesis followed was according to previous reported method[1-2] In a typical experiment, 0.5 mmol $HAuCl_4 \cdot 3H_2O$ and 2.0 mmol GSH powder were mixed in 20 mL of acetone at room temperature under vigorous stirring for 20 min. The mixture was cooled in ice bath to 0 °C and kept under stirring for 20 min. Then, a solution of $NaBH_4$ (5 mmol, dissolved in 6 mL of cold miliQ water) was added all in once under vigorous stirring. The colour of the solution immediately turned black in the reduction step with $NaBH_4$, denoting the formation of the cluster. The black $Au_n(SG)_m$ nanoclusters formed precipitated and differentiated from the acetone clear solution. This was decanted and 6 mL of water was added to dissolve the $Au_n(SG)_m$ clusters. The obtained Aun(SG)m were reacted in excess of $PhC_2H_4SH$. Typically, a solution of Aun(SG)m (around 250 mg, dissolved in 6 mL of nanopure water) was mixed with 0.3 mL of ethanol, 2 mL of toluene, and 2 mL of $PhC_2H_4SH$. The role of ethanol is to facilitate the phase transfer of Aun(SG)m from water to the organic phase. The solution is heated to 80 °C under air atmosphere for 40 h. In this time the size focusing occur leading to a high level monodisperse $Au_{38}(SC_2H_4Ph)_{24}$ clusters. The organic phase was washed with methanol to remove excess thiol. Then the $Au_{38}(SC_2H_4Ph)_{24}$ nanoclusters solution in THF was further purify in a SEC column.

The monodispersity of Au nanocluster was confirmed by UV-Vis and MALDI-TOF mass spectrometry (Figure S1)[2]. Secondly, the obtained $Au_{38}(SC_2H_4Ph)_{24}$ was supported on and$CeO_2$ $Al_2O_3$ (at 0.5 wt % Au) by impregnation. A solution of the $Au_{38}(SC_2H_4Ph)_{24}$ cluster in dichloromethane (DCM) and the oxide materials was stirred till the brown solution turned colorless. The supported nanocluster catalysts were collected by filtration and dried in air at 80˚C. Thermal pretreatments of the supported nanoclusters were carried out in air at 150 °C and 250 °C (10°C/min ramp).

**Characterization.** *High-angle annular dark field (HAADF)-Scanning Transmission Electron Microscopy (STEM)* was performed at 200 kV with a Tecnai G2 F20 S-TWIN microscope equipped with a field emission electron source and provided the microstructural characterization. *X-ray photoelectron spectroscopy (XPS)* measurements were performed on a UHV system equipped with a Phoibos 100 hemispherical analyzer and a XR 50 X-ray source (SPECS GmbH). Spectra were recorded with AlKα radiation and data were analyzed with the CasaXPS software. Peaks were fitted after Shirley background subtraction with Gauss−Lorentz sum functions. Peak positions and full width at half-maximum (FWHM) were left unconstrained. Au4f peaks were fitted with 3.7 eV doublet separation and a fixed ratio of 4:3 for Au4f7/2 and Au4f5/2. For the S2p peak fitting doublets with a fixed doublet separation of 1.2 eV and a fixed area ratio of 2:1 were used for S2p3/2 and S2p1/2 (all NIST XPS database). For the XPS measurements the powders were applied evenly on carbon tape. Peak positions were referenced to the C1s (graphite) and valence band signal.

## Supporting Figure

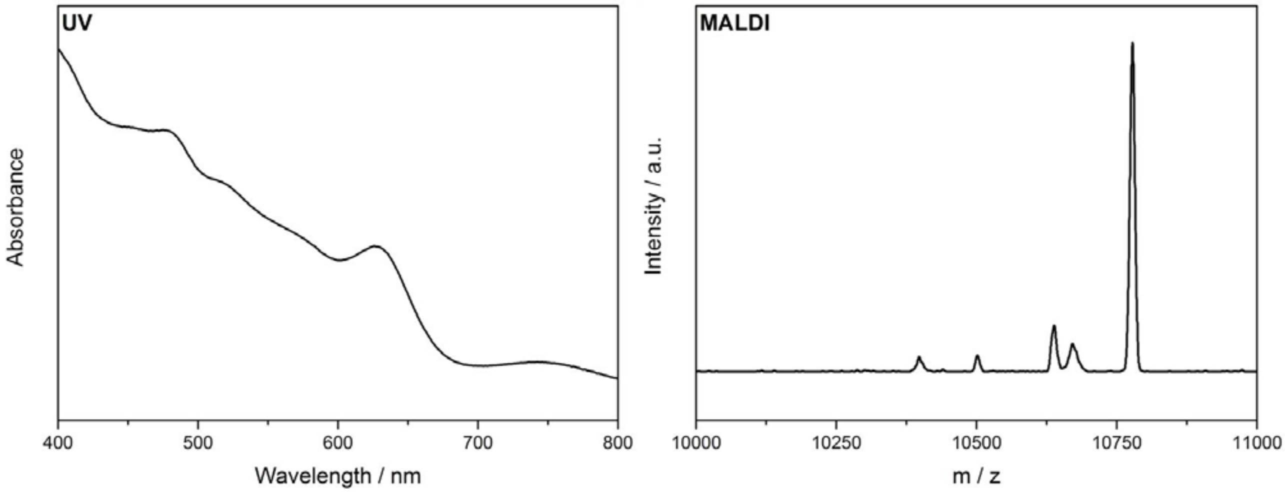


**Figure S1.** UV-Vis and MALDI of $Au_{38}(SC_2H_4Ph)_{24}$ synthesized nanoclusters.

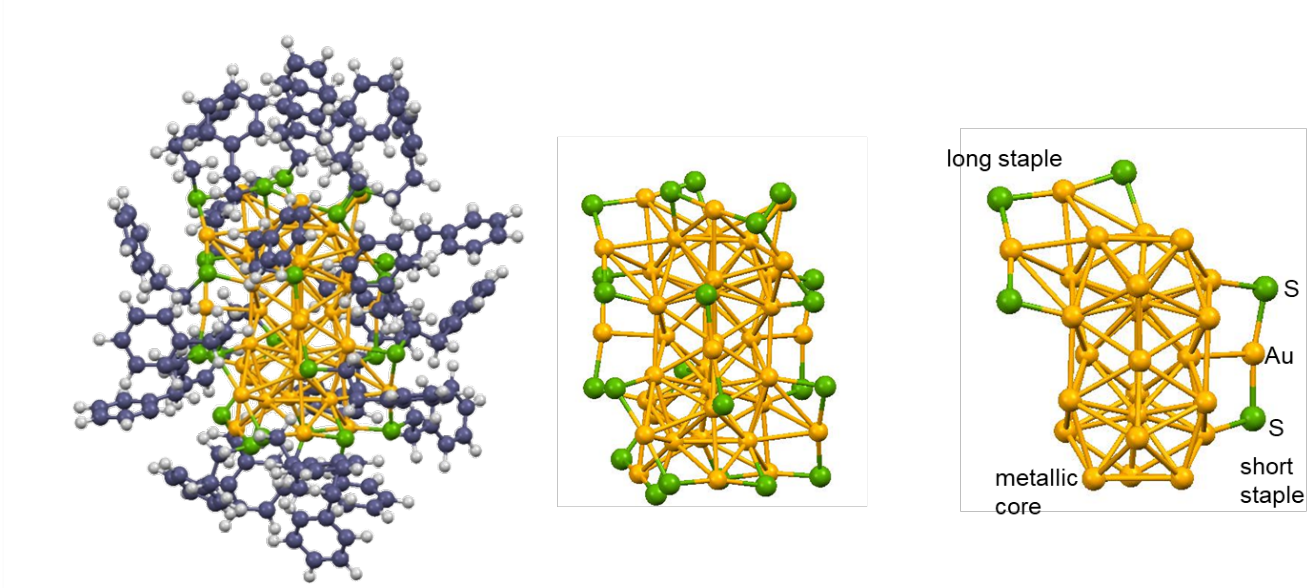


**Figure S2.** Structure of $Au_{38}(SC_2H_4Ph)_{24}$ synthesized nanoclusters.

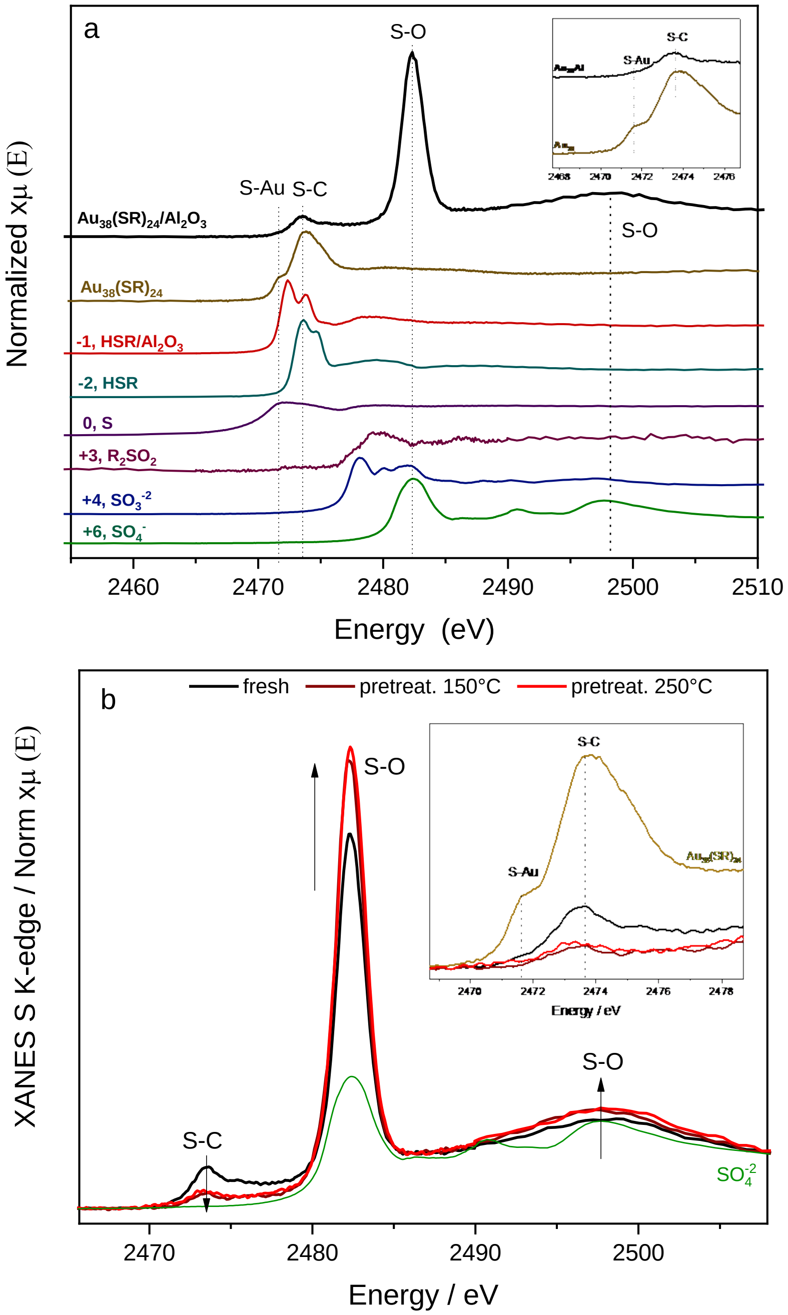


**Figure S3.** XANES spectra at S K-edge of a: $Au_{38}(SR)_{24}$ cluster (SR=$SC_2H_4Ph$) supported on $Al_2O_3$ samples with reference materials spectra; b: supported sample after pretreatment under oxygen at different temperatures.

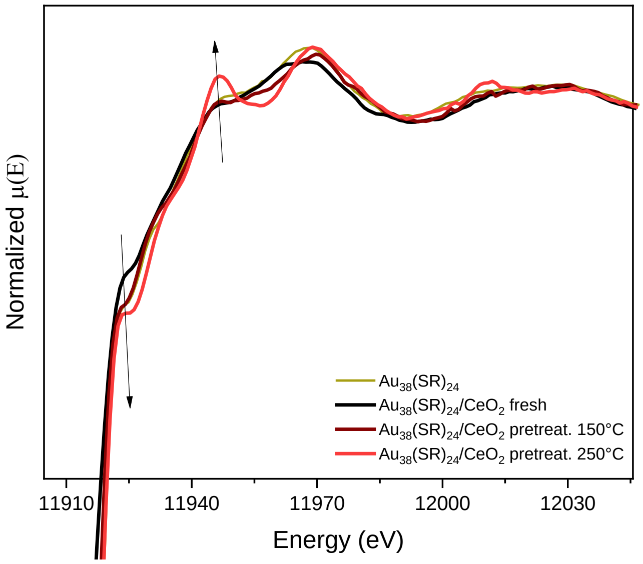


**Figure S4.** XANES spectra at Au $L_3$-edge of $Au_{38}/CeO_2$ catalysts fresh and pretreated at 150°C and 250°C under oxygen atmosphere.

Figure S4 displays the Au $L_3$-edge XANES spectra of the $Au_{38}(SR)_{24}/CeO_2$ catalysts before and after pretreatment., by probing transitions of Au 2p to unoccupied 5d states, which is correlated with the Au-thiolate bonding. From IFEFF calculations, it was concluded that the staple (non-metallic) Au site shows more pronounced d-electron depletion (and thus more intense white line) than the (metallic) core Au site.[3-4] By increasing the pre-treatment temperature, the white line (≈11920eV) decreased and shifted, indicating a more pronounced metallic state. The decrease of Au-S bonds during pretreatment also affects the peak around 11950eV leading to higher metallic character. Note, that the Au $L_3$-edge XANES of the freshly deposited sample already shows some signal around 11950eV, which indicates migration of thiolates from the gold cluster and redistribution on the support

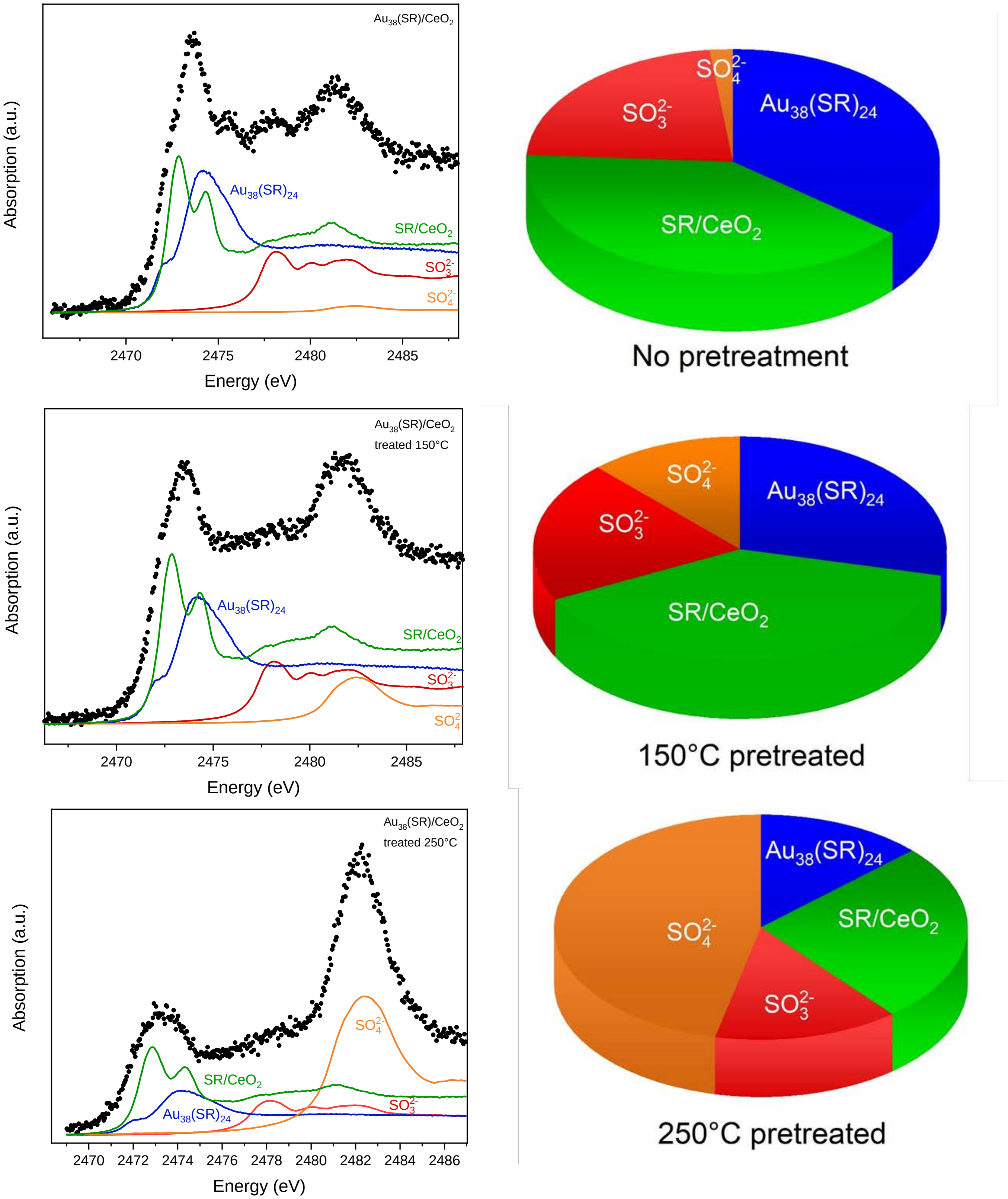


**Figure S5.** Linear Combination of S K-edge spectra of $Au_{38}(SC_2H_4Ph)_{24}$ / CeO2 without and with thermal treatment (150°C and 250°C under air), with the reference samples.

## Author Contributions

BZ, AS, GS and NB prepared the samples and preformed the basic characterization. BZ, AS, GS, SP, WO and NB prepared and performed the XAFS experiments. NB and WO analyzed the XAFS data. JL measured and analyzed the HAADF STEM and EDX. VT and CR measured and analyzed the XPS. TB and NB designed the work. BZ, TB and NB wrote the manuscript. AS, GS and GR help with the writing and revision of the manuscript.

1. Zhang, B.; Salassa, G.; Burgi, T., Silver migration between Au-38(SC2H4Ph)(24) and doped AgxAu38-x(SC2H4Ph)(24) nanoclusters. *Chemical Communications* **2016,** *52* (59), 9205-9207.
2. Zhang, B.; Kaziz, S.; Li, H.; Hevia, M. G.; Wodka, D.; Mazet, C.; Burgi, T.; Barrabes, N., Modulation of Active Sites in Supported Au-38(SC2H4Ph)(24) Cluster Catalysts: Effect of Atmosphere and Support Material. *Journal of Physical Chemistry C* **2015,** *119* (20), 11193-11199.
3. Chevrier, D. M.; Yang, R.; Chatt, A.; Zhang, P., Bonding properties of thiolate-protected gold nanoclusters and structural analogs from X-ray absorption spectroscopy. *Nanotechnology Reviews* **2015,** *4* (2), 193-206.
4. Zhang, P., X-ray Spectroscopy of Gold-Thiolate Nanoclusters. *Journal of Physical Chemistry C* **2014,** *118* (44), 25291-25299.
